# Prolactin signaling through the short isoform of the mouse prolactin receptor regulates DNA binding of specific transcription factors, often with opposite effects in different reproductive issues

**DOI:** 10.1186/1477-7827-7-87

**Published:** 2009-08-24

**Authors:** Y Sangeeta Devi, Aurora Shehu, Julia Halperin, Carlos Stocco, Jamie Le, Anita M Seibold, Geula Gibori

**Affiliations:** 1 Department of Physiology and Biophysics, College of Medicine, University of Illinois at Chicago, Illinois 60612, USA; 2 Universidad Maimonides, Hidalgo 775 – C.P.: C1405BCK, Ciudad Autonoma de Buenos Aires, Argentina

## Abstract

**Background:**

It has been well established that prolactin (PRL) signals through the long form of its receptor (PRL-RL) and activates the Jak/Stat pathway for transcription of PRL target genes. However, signaling pathways mediated through the short PRL-R isoform (PRL-RS) remains controversial. Our recent finding that PRL signaling through PRL-RS represses two transcription factors critical for follicular development lead us to examine other putative PRL/PRL-RS target transcription factors in the decidua and ovary, two well-known target tissues of PRL action in reproduction.

**Methods:**

In this investigation we used mice expressing PRL-RS on a PRL-R knockout background and a combo protein/DNA array to study the transcription factors regulated by PRL through PRL-RS only.

**Results:**

We show that PRL activation of the PRL-RS receptor either stimulates or inhibits the DNA binding activity of a substantial number of transcription factors in the decidua as well as ovary. We found few transcription factors to be similarly regulated in both tissues, while most transcription factors are oppositely regulated by PRL in the decidua and ovary. In addition, some transcription factors are regulated by PRL only in the ovary or only in the decidua. Several of these transcription factors are involved in physiological pathways known to be regulated by PRL while others are novel.

**Conclusion:**

Our results clearly indicate that PRL does signal through PRL-RS in the decidua as well as the ovary, independently of PRL-RL, and activates/represses transcription factors in a tissue specific manner. This is the first report showing PRL/PRL-RS regulation of specific transcription factors. Many of these transcription factors were not previously known to be PRL targets, suggesting novel physiological roles for this hormone.

## Background

PRL is a polypeptide hormone known to exert a great number of biological functions by regulating transcription of genes involved in several physiological pathways [rev in [1]]. PRL relays its effect by binding to specific receptors (PRL-R) and activating intracellular signaling molecules. The PRL-R belongs to type I transmembrane receptor family and structurally resembles the class I cytokine receptor superfamily [rev in [2]]. Multiple isoforms of membrane-bound PRL-R, resulting from alternative splicing of the primary transcript, have been identified in several species [3-5]. The two major PRL-R isoforms described in rodents are the short (PRL-RS) and long (PRL-RL) forms. These different PRL-R isoforms share a common extracellular domain, but differ in the length and composition of their cytoplasmic domain, that mediates signaling. The long isoform contains the entire spectrum of signaling entities attributed to PRL-R, which include Box1 and 2 motifs with the variable box (V-box) in between and an extended Box 2 (X-box) [rev in [6]]. Jak2 kinase is constitutively associated with Box 1 of the PRL-R and is rapidly activated upon ligand binding [5,7,8]. Well-known targets of activated Jak2 include the Stat transcription factors, which are primary signaling mediators of PRL action [1,7]. It was thought that PRL does not signal though PRL-RS and that this receptor serves as a dominant negative to PRL-RL, since it lacks the cytoplasmic domain required for association with signaling molecules, such as the Stat transcription factors [9-11]. However, recent data from our laboratory [12,13] and that of others [14,15], have revealed that PRL has distinct physiological functions in mice and cells expressing solely the PRL-RS. Whereas mice expressing PRL-RS display early follicular recruitment followed by severe follicular death and premature ovarian failure [13], overexpression of PRL-RS rescues the mammopoiesis defect in heterozygous PRLR knockout mice [14]. Among the human short isoforms, SF1b appears to be the closer to the mouse isoform (PR-1), as both utilize exon 11 and exclude exon 10 and both exerts dominant-negative effects on signaling by the long form. Using cell culture studies, Walker and group have shown that the human short isoform SF1b, not only acts as a dominant negative to the long form, but also induces cellular differentiation [15]. Recently, we have demonstrated both in vivo and in vitro [12,13], that PRL signaling through PRL-RS represses the activity of two transcription factors leading to inhibition in the activity of genes essential for normal follicular development.

While these studies show a distinct physiological function of PRL-RS, the signaling mechanisms involved in these processes remain largely unidentified. Since one major end-point of hormone signaling is activation/deactivation of transcription factors, detection of the transcription factors regulated specifically after PRL-RS activation can provide important information on PRL action through PRL-RS. Traditionally, transcription factor activity has been analyzed by electrophoretic mobility shift assay (EMSA) or by detection of specific posttranslational modifications known to affect DNA binding activity, such as phosphorylation. Unfortunately, these methods can detect activation of only one transcription factor/reaction and also require a large amount of sample. Here, we used a high throughput protein/DNA array approach in which the activities of a large number of transcription factors can be easily detected using biotin-labeled DNA binding probes and a chemiluminescence method. We took advantage of this array and the availability of transgenic mice expressing only PRL-RS in the PRLR null background [13], which allow us to study the selective signaling mechanism of PRL-RS independently of PRL-RL, in vivo. By combining these two powerful models, we identified several transcription factors regulated by PRL/PRL-RS. Our results reveal putative novel mechanisms of PRL signaling in two important PRL target tissues, the decidua and ovary.

## Methods

### Animal model and tissue preparation

Transgenic mice expressing only PRL-RS were previously generated by microinjecting the eF1-PRLR-PR-1 transgenic construct encoding the mouse cDNA for PRL-RS into fertilized PRLR^+/- ^oocytes derived from 129SV pure background mice [14]. Animals were genotyped by PCR using genomic DNA isolated from tail as described previously [13]. Mice were kept at 25°C with a 14-h light/10-h dark cycle and were fed a commercial pellet diet ad libitum.

Female PRL-RS transgenic mice were mated with vasectomized males to induce pseudopregnancy. Progesterone pellet (25 mg, Innovative Research of America, Sarasota, FL) was implanted sc in each mouse on the day when the vaginal plug was found. Decidualization was induced with intrauterine administration of sesame oil on day 4. On day 9 of pseudopregnancy, mice were injected with ergocryptine (200 ug, s.c., Sigma, St. Louis, MO) to block PRL secretion. After 6 hrs, a single i.p. injection of either recombinant oPRL (60 ug) purchased from Dr. Arieh Gertler (Protein Laboratories Rehovot Ltd., Rehovot, Israel) or vehicle (0.1%BSA in PBS) was given to the mice. Mice were sacrificed after 30 and 120 min to obtain ovaries and decidua that were frozen in liquid nitrogen and stored at -80°C until processing for RNA or protein extraction. All experimental procedures were performed in accordance with the principles of the National Institutes of Health Guide for the Care and Use of Laboratory Animals and were approved by the Institutional Animal Care and Use Committee.

### Protein/DNA array

Nuclear extracts from decidual and ovarian tissue were prepared as previously described [16]. Three animals were used for each time point. Activities of transcription factors were screened using six different TranSignal protein/DNA arrays according to the manufacturer's instructions (Panomics, Inc., Redwood City, CA, USA). In brief, the nuclear extracts were incubated with a set of biotin-labeled DNA binding oligonucleotides to allow the formation of protein/DNA complexes. The protein/DNA complexes were separated from the free probes, and the oligonucleotides in the complexes were isolated from the proteins and hybridized to the TranSignal (combo) Array. This array contains consensus binding site for 345 transcription factors as individual spot. Detection of the signals was obtained by chemiluminescence. The signal was scanned with a phosphorImager (Molecular Dynamics, CA) and spot intensities were analyzed using the AtlasImage 1.5 software (Clontech, CA). Transcription factors were excluded if they were detected at levels near or below background. Differences over 2-fold in the intensity of the spots were considered significant.

### Electrophoresis Mobility Shift Assay

Five picomoles of RUNX1 annealed oligonucleotide probes are labeled using 10 U of T4 polynucleotide kinase (Invitrogen, Carlsbad, CA) and 25 μCi of γ-^32^P ATP (Amersham, Piscataway, NJ) to a specific activity of more than 8000 cpm/fmol. Five micrograms of nuclear extract are incubated with 1 μg of poly (dI-dC) (Amersham) and 50 fmol of probe in 1× binding buffer on ice for 30 min. Cold competitor probes are added to a final concentration of 2.5 pmol. Samples are run on a 4% nondenaturing polyacrylamide gel in 0.5× Tris borate EDTA buffer at 200 V for 2–3 h. The gels are then dried and analyzed by autoradiography.

## Results

### Regulation of Transcription factor activities by PRL in the ovary and decidua of transgenic mice expressing PRL-RS

**Figure 1:**
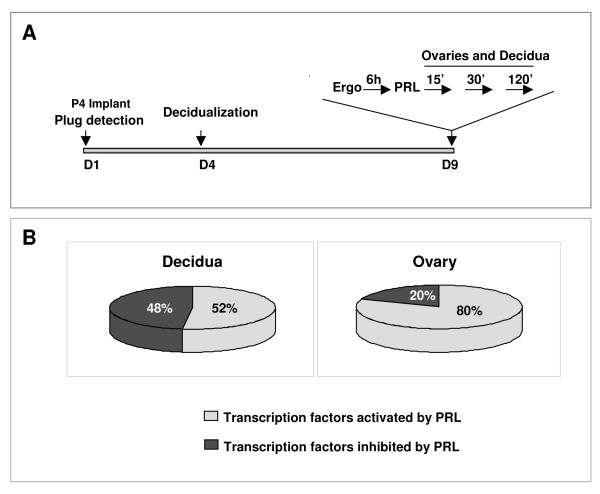
**Animal model and DNA/protein array analysis in the PRLR-/-RS mice**. (A) PRLR-/-RS mice were mated with vasectomized males to induce pseudopregnancy. The day the vaginal plug is found, PRLR-/-RS mice were implanted with progesterone pellets. On day 4, decidualization was induced with intrauterine administration of oil. On day 9 of pseudopregnancy, PRLR-/-RS mice were injected with ergocryptine (200 ug, sc) for 6 hr followed by a single ip injection of either PRL (60 ug) or vehicle (control). Ovaries and decidua were obtained after 0, 30 and 120 min of PRL treatment. (B) TranSignal, Protein/DNA array, was performed using nuclear extracts from these tissues. Chart showing percentage of transcription factors activated or inhibited in decidua and ovary.

To determine the downstream signaling pathways of PRL/PRL-RS, we examined the activities of transcription factors in the ovary and decidua of PRL-RS expressing mice using the TranSignal protein/DNA array. For this purpose (Fig. 1A), decidualization was induced with an intrauterine oil injection in day 4 pseudopregnant transgenic mice expressing only PRL-RS. On day 9, mice were treated with ergocryptine, to block endogenous PRL secretion, followed by PRL injections. Decidua and ovaries were harvested after 30 and 120 min of PRL administration and nuclear extracts were isolated. Out of 345 transcription factors analyzed, 40 were found to be regulated by PRL in the decidua, while 66 were regulated in the ovary, as shown by DNA binding activity. In the decidua (Fig. 1B), 52% of the regulated transcription factors were activated whereas, 48% were inhibited by PRL. In the ovary (Fig. 1B), only few transcription factors (20%) were inhibited, whereas the majority (80%) of the DNA binding proteins was activated. The transcription factors that are not regulated by PRL in the decidua [see additional file 1] and the ovary [see Additional file 2] are also listed.

**Figure 2:**
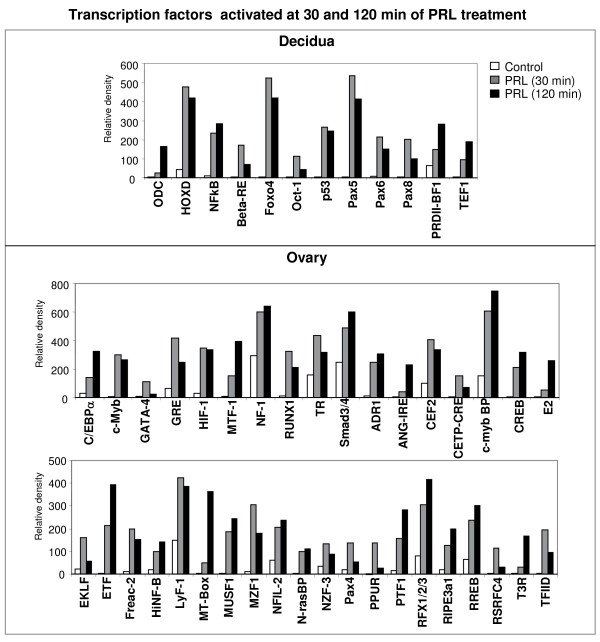
**Transcription factors activated at both 30 and 120 min of PRL treatment in decidua and ovary of PRLR-/-RS mice**. Day 9 pseudopregnant PRLR-/-RS mice were injected with ergocryptine (200 μg, sc) for 6 hr followed by a single ip injection of either PRL (60 ug) or vehicle (control). Decidua and ovaries were obtained after 0, 30 and 120 min of PRL treatment. DNA binding activities of transcription factors were measured by Protein/DNA binding assay using nuclear extracts from decidua. Upper panel, transcription factors activated by PRL in the decidua at both 30 and 120 min. Lower panel, transcription factors activated by PRL in the ovary at both 30 and 120 min.

Next, we categorized transcription factors as those either activated or inhibited by PRL in the decidua and ovary at early (30 min) and late (120 min) time points. As shown in Fig. 2, fewer transcription factors were activated at both time points in the decidua (upper panel) than in the ovary (lower panel). Interestingly, the transcription factor that regulates ornithine decarboxylase (ODC), the best established marker of decidualization, was activated in the decidua, suggesting a role for PRL/PRL-RS in the expression of this important decidual gene. NFkB, known to be activated by PRL in other tissues [17,18], was also activated in the decidua, suggesting a broad role of PRL in the activation of these DNA binding proteins. Interestingly, we found other transcription factors, such as Foxo4, Oct-1 and several members of the Pax family, not previously known to be expressed in the decidua or to be regulated by PRL, to be robustly activated by PRL in decidua expressing the short form of the PRL receptor, indicating new PRL signaling mechanisms in this tissue. In the ovary, a much greater number of transcription factors were activated at 30 min of PRL treatment, and remained activated at 120 min (Fig. 2, lower panels). Many of these transcription factors such as C/EBPα, c-Myb, GATA-4, GRE, HIF-1, MTF, NF-1, RUNX1/PEBP2, TR, and Smad3/4 were previously shown to be expressed in the ovary and to regulate genes involved in ovarian steroidogenesis and function [19-26]. However, our finding that they are robustly activated by PRL through PRL-RS is novel. This is the first report of the expression and PRL mediated activation of ovarian DNA binding proteins (Fig. 2, lower panel).

**Figure 3:**
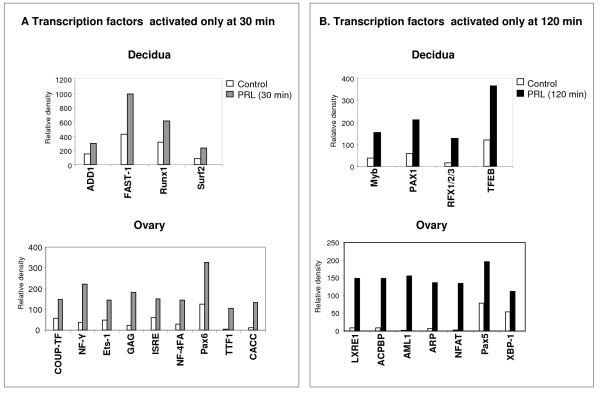
**Transcription factors activated transiently either at 30 min or 120 min by PRL treatment in decidua and ovary of PRLR-/-RS mice**. Day 9 pseudopregnant PRLR-/-RS mice were injected with ergocryptine (200 μg, sc) for 6 hr followed by a single ip injection of either PRL (60 ug) or vehicle (control). Decidua and ovaries were obtained after 0, 30 and 120 min of PRL treatment. DNA binding activities of transcription factors were measured by Protein/DNA binding assay using nuclear extracts from ovary. (A) Transcription factors activated at 30 min by PRL treatment in the decidua (upper panel) and ovary (lower panel). (B) Transcription factors activated at 120 min by PRL treatment in the decidua (upper panel) and ovary (lower panel).

As shown in Fig. 3, some of the DNA binding proteins were activated only transiently either at 30 min or 120 min after PRL treatment. In the decidua, we found four transcription factors to be activated at 30 min (Fig. 3A, upper panel) or 120 min (Fig. 3B, upper panel) of PRL treatment. The expression and PRL activation of these transcription factors in the decidua have not been previously reported. In the ovary, nine transcription factors were found to be activated only at 30 min (Fig. 3A, lower panel). Notable transcription factors in this category, such as COUP-TF and NF-Y, were previously shown to play a distinct role in ovarian functions [27-29], whereas Ets-1 has been reported in the normal ovary as well as in pathological conditions [30,31]. Whether PRL/PRL-RS activation of this transcription factor plays a role in the pathology of the ovary remains a subject of investigation. Interestingly, a few of the transcription factors such as Myb and RFX1/2/3, which were activated early in the ovary (Fig. 2C, upper panel), showed delayed activation in the decidua (Fig. 3B, upper panel). Most of the ovarian transcription factors activated at 120 min (Fig. 3B, lower panel) such as AML1, NFAT and Pax5 are known to be involved in the immune response.

**Figure 4:**
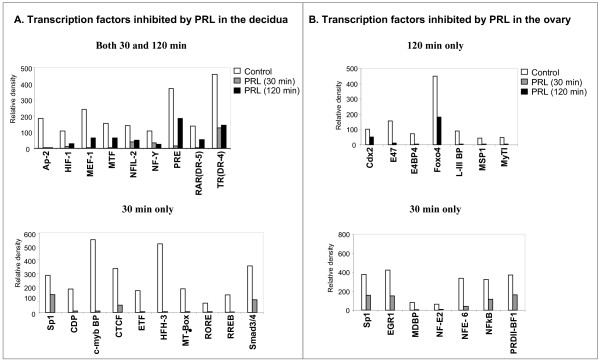
**Transcription factors inhibited by PRL treatment in decidua and ovary of PRLR-/-RS mice**. Day 9 pseudopregnant PRLR-/-RS mice were injected with ergocryptine (200 μg, sc) for 6 hr followed by a single ip injection of either PRL (60 ug) or vehicle (control). Decidua and ovaries were obtained after 0, 30 and 120 min of PRL treatment. DNA binding activities of transcription factors were measured by Protein/DNA binding assay using nuclear extracts from decidua. (A) Transcription factors whose DNA binding activities are inhibited at both time points (upper panel) or inhibited only at 30 min (lower panel) by PRL treatment in decidua. (B) Transcription factors whose DNA binding activities are inhibited at 120 (upper panel) or 30 min (lower panel) by PRL treatment in ovary.

We also analyzed the transcription factors repressed by PRL/PRL-RS (Fig. 4). In the decidua of PRL-RS expressing mice, nine transcription factors were profoundly repressed by PRL at both 30 and 120 min (Fig. 4A, upper panel), while ten were repressed only at 30 min (Fig. 4A, lower panel). Only few of these transcription factors, such as Sp1, HIF-1, NF-Y, PRE and RAR, have known functions in the decidua [32-35]. In the ovary, no transcription factors were repressed at both 30 and 120 min, but different sets of transcription factors were markedly inhibited at either 120 min (Fig. 4B, upper panel), or 30 min (Fig. 4B, lower panel). Sp1, EGR1 and NFKB, three known targets of PRL [12,18,36], were also inhibited in the ovary (Fig. 2D, lower panel). Interestingly, Cdx2 and E47, known to be upregulated in ovarian carcinoma or PCOS ovaries, were repressed in the ovary at 120 min by PRL/PRL-RS signaling (Fig. 4b, upper panel).

### Differential regulation of transcription factors in decidua and ovary by PRL through PRL-RS

Surprisingly, we found only six transcription factors similarly regulated by PRL in the ovary as well as in the decidua of PRL-RS expressing mice (Table 1). Whereas Pax5, Pax6, RUNX1 and RFX activities were stimulated by PRL in both target tissues, cdx2 and Sp1 activities were inhibited. Pax5 was one of most highly activated transcription factor in the decidua (Fig. 2A).

**Table 1:** Transcription factors similarly regulated by PRL in the decidua and ovary of PRLR-/-RS mice

*Transcription Factor ID*	*Decidua*	*Ovary*
Pax5	Activated	Activated
Pax6	Activated	Activated
RUNX1	Activated	Activated
RFX1/2/3	Activated	Activated
Cdx2	Inhibited	Inhibited
Sp1	Inhibited	Inhibited

**Figure 5:**
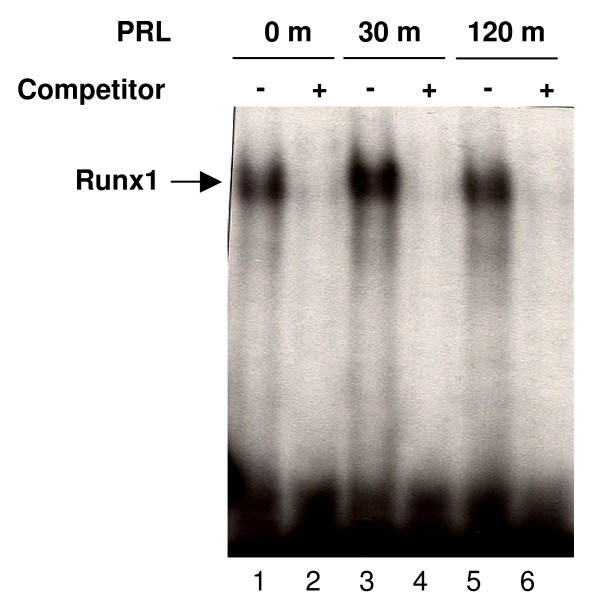
**DNA binding activity of RUNX1**. Day 9 pseudopregnant PRLR-/-RS mice were injected with ergocryptine (200 μg, sc) for 6 hr followed by a single ip injection of either PRL (60 ug) or vehicle (control). Decidua were obtained after 0, 30 and 120 min of PRL treatment. Nuclear extract from decidua was subjected to EMSA using oligonucleotide specific probe to RUNX1. Lane 1, 3 and 5 are nuclear extracts isolated from decidua of 0, 30 and 120 min PRL treated animals, respectively. Lane 2, 4 and 5 are nuclear extracts treated with competitor, a 50-fold molar excess of unlabeled probe.

RUNX1 is another transcription factor whose activity was modulated similarly in the decidua and ovary. This transcription factor was transiently activated at 30 min in decidua while it remained activated in the ovary for a longer time. We further examined the regulation of RUNX1 in the decidual nuclear extracts from mice expressing only PRL-RS by EMSA analysis. A double-stranded DNA oligo corresponding to RUNX1 binding sites was used as the probe. We found a single DNA/protein complex that was markedly increased by PRL treatment at 30 min only (Fig. 5). This complex could be blocked by competition with excess cold probe confirming the specificity of the band.

In sharp contrast, we found a significant number of transcription factors, including Foxo4, NFkB, NFY and Smad3/4, to be inhibited in the decidua and to be stimulated in the ovary suggesting that PRL may activate/deactivate similar transcription factors through PRL-RS in an opposite way depending on the target tissue (Table 2).

**Table 2:** Transcription factors oppositely regulated by PRL in the decidua and ovary of PRLR-/-RS mice

*Transcription Factor ID*	*Decidua*	*Ovary*
NFkB	Activated	Inhibited
Foxo4	Activated	Inhibited
NF-Y	Inhibited	Activated
Samd3/4	Inhibited	Activated
ETF	Inhibited	Activated
HIF-1	Inhibited	Activated
ISER	Inhibited	Activated
MT-Box	Inhibited	Activated
MTF	Inhibited	Activated
NFIL-2	Inhibited	Activated
PRDII-BF1	Activated	Inhibited
RREB	Inhibited	Activated
c-myb BP	Inhibited	Activated
TR	Inhibited	Activated

### Functional category of PRL regulated transcription factors

Although some transcription factors have redundant action, we grouped them (Table 3) according to the function of the genes whose transcription they regulate. These functional categories are growth and differentiation, development and steroidogenesis, immune, inflammatory and stress response, and glucose metabolism.

**Table 3:** List of transcription factors by functional category

**A. Growth and Differentiation**			
*Decidua*		*Ovary*	

Myb	Activated	C/EBPalpha	Activated
ODC	Activated	c-Myb	Activated
Pax1	Activated	EGR1	Inhibited
RUNX1	Activated	HIF-1	Activated
PRDII-BF1	Activated	NZF-3	Activated
RFX1/2/3	Activated	TFIID	Activated
TEF1	Activated		
TFEB	Activated		
AP-2	Inhibited		
c-Myb BP	Inhibited		
HIF-1	Inhibited		
MT-Box	Inhibited		
MTF-1	Inhibited		
RAR (DR-5)	Inhibited		

**B. Development and Steroidogenesis**			

*Decidua*		*Ovary*	

CTCF	Inhibited	Cdx2	Inhibited
HFH-3	Inhibited	COUP-TF	Activated
HOXD8,9,10	Activated	EKLF	Activated
Pax6	Activated	GATA-4	Activated
Pax8	Activated	MZF1	Activated
		Pax4	Activated
		Pax6	Activated

**C. Immune Response**			

*Decidua*		*Ovary*	

Pax5	Activated	AML1	Activated
CDP	Inhibited	GRE	Activated
NFIL-2	Inhibited	LyF-1	Activated
RORE	Inhibited	NFAT	Activated
		Pax5	Activated

**D. Inflammation/Stress**			

*Decidua*		*Ovary*	

Foxo4	Activated	Foxo4	Inhibited
NFkB	Activated	NFkB	Inhibited
Sp1	Inhibited	Sp1	Inhibited
		E4BP4	Inhibited

**E. Glucose Metabolism**			

*Ovary*			
		
ADR1	Activated		
ANG-IRE	Activated		
MUSF1	Activated		
PTF1	Activated		
RIPE3a1	Activated		
L-III BP	Inhibited		

In the decidua, close analysis of the array data revealed that, a majority of the transcription factors regulated by PRL is involved in growth and differentiation (Table 3A). We found Myb, ODC-TF, Pax1, PEBP2, PRDII-BF1, RFX, TEF1, and TEBP to be activated by PRL, while AP-2, c-myb BP, HIF-1, MT-box, MTF-1, and RAR/DR-5 to be inhibited. Although few of the transcription factors (ODC-TF, AP-2, c-myb, and HIF-1) are established PRL targets [23,37-40], this is the first report showing their regulation through PRL-RS. The PRL regulation of several other factors is novel and reveals unknown functions of this pleiotropic hormone in the decidua. Several transcription factors, involved in development (Table 3B), were also regulated by PRL in the decidua of PRLR-/-RS mice. These include: HOXD, Pax6, Pax8, CTCF, and HFH3. These transcription factors are not known to be regulated by PRL, although HOXD plays a key role in endometrial proliferation, which is a prerequisite event for decidualization [41]. Interestingly, three out of four of the transcription factors involved in the immune response, namely NFIL-2, CDP and RORE, were inhibited by PRL signaling through PRL-RS in the decidua (Table 3C). We also found the binding activity of a small number of transcription factors involved in inflammatory/stress response (Foxo4, NFκB, and Sp1) to be regulated by PRL in the decidua (Table 3D). PRL was shown previously to regulate Sp1 [12], NFκB [18,42] and another member of the Foxo family (Foxo3a). No transcription factor involved in glucose metabolism was regulated by PRL in the decidua.

In contrast to the decidua, a significant number of transcription factors regulated by PRL in the ovary are involved in glucose metabolism (Table 3E). This further supports our previous finding [13] indicating that PRL signaling through PRL-RS in the ovary profoundly affects glucose metabolism. We also found transcription factors involved in growth and differentiation (Table 3A), development (Table 3B), immune (Table 3C), and inflammatory/stress response (Table 3D) to be regulated by PRL in the ovary. Some of the transcription factors grouped under development such as GATA-4 [43], and COUP-TF [44] also play critical roles in steroidogenesis. Of interest was our finding that some of the transcription factors involved in the immune response, such as AML1, GRE, LyF-1, NFAT and Pax5, are activated by PRL in the ovary of transgenic mice expressing PRL-RS, whereas transcription factors involved in inflammatory or stress responses, such as NFκB, E4BP4 and Sp1 are inhibited (Table 3C and 3D).

## Discussion

We have recently provided evidence that PRL activation of PRL-RS regulates the transcriptional activity of several genes[12]. We have also shown, using cells expressing either PRL-RL or PRL-RS, that activation of PRL-RS by PRL represses the gene expression of Sp1 [12]. This indicated to us that PRL-RS is a signaling receptor and that PRL activation of this receptor leads to activation/repression of genes. Since gene regulation involves transcription factors we used a combo protein/DNA array approach and mice expressing only PRL-RS on a PRLR knockout background to analyze the role of PRL on transcription factor activity in two well established PRL target organs.

We found indeed that PRL markedly affects the activation or inhibition of DNA binding activity of a substantial number of transcription factors. Some were regulated similarly in both ovary and decidua, while others were regulated in an opposite way. The large majority however, was regulated solely in either the ovary or decidua, suggesting that PRL may affect several signaling pathways in a tissue specific manner. One possibility for these differential effects may be the cellular milieu of the target tissue such as the level of expression of specific transcription factors and/or availability of scaffolding proteins. Scaffolding proteins are known to interact with signaling molecules and prevent their degradation and/or facilitate their localization at specific sites [45].

When organized by function, it became apparent that the PRL/PRL-RS regulated transcription factors are involved in glucose metabolism, growth and differentiation, the immune response, inflammation and development and steroidogenesis. While some of these transcription factors were previously shown to be regulated by PRL [18,36,46], this is the first report of their regulation through PRL-RS. The PRL mediated activation/repression of other factors and DNA binding proteins is novel and deserves further investigation. One salient finding of this investigation is that several transcription factors involved in glucose metabolism are regulated by PRL-RS in the ovary but not at all in the decidua. It is well known that defective glucose metabolism in the ovary causes severe pathology in follicular development and premature ovarian failure in women in their twenties [47,48]. One key enzyme in gluconeogenesis, GALT (galactose-1-phosphate uridyltransferase), is abundantly expressed in the ovary. Mutation of this gene in humans causes a defect in glucose synthesis and results in an accumulation of galactose metabolite that cannot pass through the cellular membrane. Its accumulation into the cells causes an osmotic disequilibrium that leads to water influx and ultimately to cell death. We have recently shown [13] that in mice expressing only PRL-RS, PRL represses the expression of GALT and causes premature ovarian failure and non-apoptotic cell death, similar to the pathology seen in women with the GALT mutation [47-49]. We have also shown that it is the repression of Foxo3a transcription factor by PRL/PRLRS that leads to low levels of GALT [13]. The finding that a number of additional ovarian transcription factors involved in glucose metabolism are regulated by PRL through PRL-RS specifically in the ovary may be of physiological significance and deserves further investigation. Another known effect of PRL in the follicle is the inhibition of aromatase activity [50]. Our finding that PRL activates COUP-TF, a known repressor of aromatase [51], suggests a role for PRL activation of PRL-RS in the repression of this enzyme in the ovary. c-Myb, also regulated by PRL, stimulates transcription of genes involved in angiogenesis such as VEGF [52]. The induction of c-Myb by PRL through PRL-RS is of interest since PRL-RS is expressed in the vasculature and signaling through this receptor has been implicated in endothelial cell proliferation [53].

PRL is thought to play a role in modulating the immune response in utero during pregnancy [42,54]. Importantly, we find most of the transcription factors involved in the immune response to be inhibited by PRL signaling through PRL-RS in the decidua. More than 40% of decidual cells are lymphocytes [55,56] and the PRL-R is expressed by this cell type [1,57,58]. Some transcription factors regulated by PRL/PRL-RS in the decidua are known to control genes whose expression/repression plays a role in decidualization. These include activator of ODC, the transcription factor necessary for ornithine decarboxylase (ODC) expression, the retinoic acid receptor (RAR/DR-5), and members of the HOXD family. ODC, the enzyme that catalyzes the transformation of L-ornithine into putrescine, is a key regulatory member in the polyamine biosynthetic pathway. ODC activity is associated with the rate of growth [59]. Since tissue growth is an essential step in the process of decidualization, ODC is considered as marker enzyme for decidualization [60]. PRL is known to stimulate ODC expression as well as enzyme activity [23]. Our finding suggests that the PRL-mediated regulation of decidual ODC may involve PRL-RS and may be an important function of this receptor in decidua. In contrast, transcription factors such as RAR/DR-5, which has been shown to suppress decidualization are inhibited in the decidua by PRL/PRLRS [61]. Members of the HOXA and HOXD family of transcription factors are expressed in the decidua. They are involved in implantation and endometrial proliferation [41]. Whereas HOXA transcription factors are regulated by steroids, the regulation of members of HOXD family is not known [62]. The marked stimulation of HOXD DNA binding activity, by PRL, in the decidua of mice expressing PRL-RS is intriguing and opens new venues of investigation.

Another intriguing finding of this investigation is that several members of the Pax family are activated by PRL in the decidua as well as the ovary. Pax genes are regulators of tissue development and cellular differentiation in embryos, acting to promote cell proliferation, cell-lineage specification, migration and survival. The function of these Pax members in the adult ovary and decidua is not known. However, several investigations indicate that the regulation of these transcription factors involves methylation. Pax2 is silenced by hypermethylation of its promoter [63]. Separate studies have reported silencing of Pax6 by methyl-CpG-binding proteins in some breast cancer cell lines and primary tumours [64]. Pax5 is inactivated by aberrant methylation in tumor cell lines, as well as primary tumors from breast and lung [65]. The increase in the DNA binding activity of Pax proteins may be a result of increased expression due to hypomethylation in response to PRL/PRL-RS signaling. Previously, PRL has been shown to cause hypomethylation of DNA in the liver and kidney of immature and mature rats [66]. Whether PRL signaling through PRL-RS affects the DNA methylation pathway in both decidua and ovary remain a subject for further investigation.

Another transcription factor activated in both decidua and ovary is RUNX1. This protein belongs to a family of runt-related transcription factors and binds to a consensus sequence known as a PEPB2 site. RUNX1/PEBP2 is critical for hematopoiesis and plays an important role in differentiation of many cell types by recruiting corepressors and coactivaotors [67]. Most importantly, RUNX1 has been shown to regulate the expression of extracellular matrix proteins, MMPs and TIMPs [68,69], which are essential for decidual as well as corpus luteum maintenance and function [rev in [70]]. Moreover, recent investigations have shown an important role for RUNX1 in luteinization and corpus luteum formation [71,72]. Whether PRL plays a role in this process by activating RUNX1 is an interesting possibility.

## Conclusion

In conclusion, we have found that DNA binding activities of several transcription factors are either activated or inhibited by PRL in the decidua and ovary of PRLR-/-RS mice. Transcription factors, such as the Pax family and RUNX1/PEBP2, are similarly regulated in both decidua and ovary, suggesting that some of the PRL/PRL-RS signaling pathways are common to both of these reproductive tissues. However, several transcription factors are differentially regulated in the decidua and ovary by PRL/PRL-RS, suggesting tissue specific signaling pathways and gene expression. Overall, our results have suggested several possible mechanisms of PRL signaling through PRL-RS in two reproductive tissues. Further studies should reveal the specific signaling pathways involved in the regulation of these transcription factors and their downstream genes.

## Competing interests

The authors declare that they have no competing interests.

## Authors' contributions

YSD performed experiments on ovarian tissue, analysis and interpretation of data and manuscript drafting. AS performed experiments on decidual tissue, analysis and interpretation of data. JH participated in maintenance of transgenic mice colony. CS participated in designing the study. JL and AMS participated in many aspects of this work. GG conceived the idea of the study, participated in its design, data analysis and drafted the manuscript. All authors read and approved the final manuscript.

## Supplementary Material

Additional file 1 **Table 4**. Transcription factors not regulated by PRL in the decidua of PRLR-/-RS mice. Click here for file

Additional file 2 **Table 5**. Transcription factors not regulated by PRL in the ovary of PRLR-/-RS mice. Click here for file
